# Highly Variable Pharmacokinetics of Tyramine in Humans and Polymorphisms in OCT1, CYP2D6, and MAO-A

**DOI:** 10.3389/fphar.2019.01297

**Published:** 2019-10-30

**Authors:** Muhammad Rafehi, Frank Faltraco, Johannes Matthaei, Thomas Prukop, Ole Jensen, Aileen Grytzmann, Felix G. Blome, Ralf Günter Berger, Ulrich Krings, Stefan V. Vormfelde, Mladen V. Tzvetkov, Jürgen Brockmöller

**Affiliations:** ^1^Institute of Clinical Pharmacology, University Medical Center Göttingen, Georg-August University, Göttingen, Germany; ^2^Institute of Food Chemistry, Leibniz University, Hannover, Germany

**Keywords:** biogenic amine, CYP2D6, MAO, monoamine oxidase, OCT1, pressor response, SLC22A1, tyramine

## Abstract

Tyramine, formed by the decarboxylation of tyrosine, is a natural constituent of numerous food products. As an indirect sympathomimetic, it can have potentially dangerous hypertensive effects. *In vitro* data indicated that the pharmacokinetics of tyramine possibly depend on the organic cation transporter OCT1 genotype and on the CYP2D6 genotype. Since tyramine is a prototypic substrate of monoamine oxidase A (MAO-A), genetic polymorphisms in MAO-A may also be relevant. The aims of this study were to identify to what extent the interindividual variation in pharmacokinetics and pharmacodynamics of tyramine is determined by genetic polymorphisms in OCT1, CYP2D6, and MAO-A. Beyond that, we wanted to evaluate tyramine as probe drug for the *in vivo* activity of MAO-A and OCT1. Therefore, the pharmacokinetics, pharmacodynamics, and pharmacogenetics of tyramine were studied in 88 healthy volunteers after oral administration of a 400 mg dose. We observed a strong interindividual variation in systemic tyramine exposure, with a mean AUC of 3.74 min*µg/ml and a high mean CL/F ratio of 107 l/min. On average, as much as 76.8% of the dose was recovered in urine in form of the MAO-catalysed metabolite 4-hydroxyphenylacetic acid (4-HPAA), confirming that oxidative deamination by MAO-A is the quantitatively most relevant metabolic pathway. Systemic exposure of 4-HPAA varied only up to 3-fold, indicating no strong heritable variation in peripheral MAO-A activity. Systolic blood pressure increased by more than 10 mmHg in 71% of the volunteers and correlated strongly with systemic tyramine concentration. In less than 10% of participants, individually variable blood pressure peaks by >40 mmHg above baseline were observed at tyramine concentrations of >60 µg/l. Unexpectedly, the functionally relevant polymorphisms in OCT1 and CYP2D6, including the CYP2D6 poor and ultra-rapid metaboliser genotypes, did not significantly affect tyramine pharmacokinetics or pharmacodynamics. Also, the MOA-A genotypes, which had been associated in several earlier studies with neuropsychiatric phenotypes, had no significant effects on tyramine pharmacokinetics or its metabolism to 4-HPAA. Thus, variation in tyramine pharmacokinetics and pharmacodynamics is not explained by obvious genomic variation, and human tyramine metabolism did not indicate the existence of ultra-low or -high MAO-A activity.

## Introduction

The biogenic amine tyramine is formed through decarboxylation of tyrosine by microbes, and is thus found in numerous fermented, aged, or ripened protein-rich food products (McCabe-Sellers et al., 2006; Ladero et al., 2010; Gillman, 2018). Tyramine functions as an indirect sympathomimetic by promoting the release of noradrenaline from synaptic vesicles, thereby having hypertensive effects (referred to as the tyramine pressor response; Gillman, 2018). In combination with monoamine oxidase (MAO) inhibitors, tyramine can cause a so-called hypertensive crisis that, beside symptoms like headache, migraine, nausea, or vomiting, may even cause end-organ damage, intracerebral haemorrhage, and death (Blackwell, 1963; Gillman, 2018; Salter and Kenney, 2018). Nowadays, potential new drugs known or suspected to inhibit MAO are usually assessed for potential interactions with oral tyramine in clinical studies.

**Figure 1:**
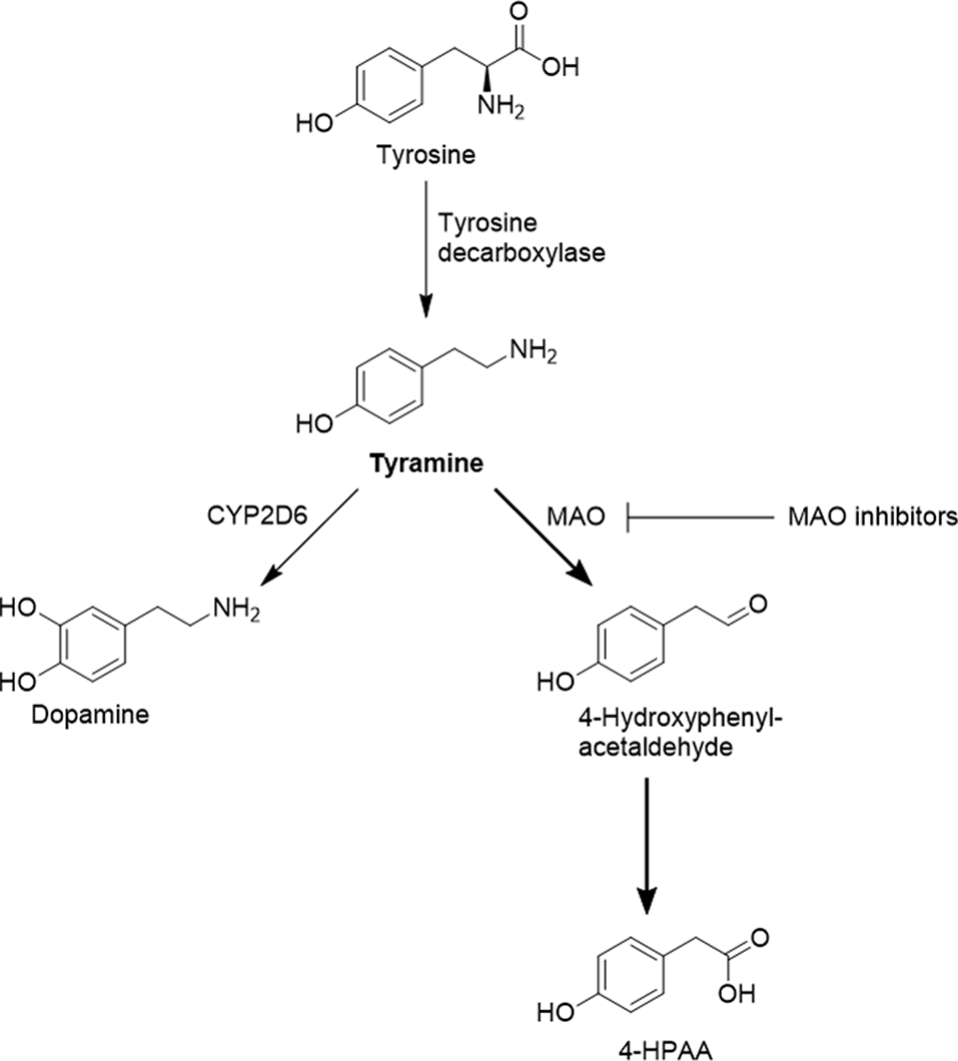
Selected pathways of tyramine formation and metabolism. Prior to oxidative deamination by MAO-A, tyramine has to traverse the cell membrane, most likely supported by organic cation transporters such as OCT1.

Genomic variation in tyramine pharmacokinetics and pharmacodynamics has thus far only been scarcely studied. MAOs catalyse the oxidative deamination of tyramine to 4-hydroxyphenylacetaldehyde, which is then converted to 4-hydroxyphenylacetic acid (4-HPAA; **Figure 1**). Tyramine was the first known substrate for MAO, which had initially been referred to as tyramine oxidase (Hare, 1928), and may still today be a suitable *in vivo* probe drug for studying MAO activity. Although tyramine is a substrate of both MAO-A and MAO-B, the tyramine pressor response occurs only with MAO-A inhibition (Finberg and Tenne, 1982; Finberg, 2014). MAO-A in the intestinal mucosa is likely to be responsible for the low oral bioavailability of tyramine, and systemic tyramine is eliminated *via* hepatic MAO (Gillman, 2018). Literature data on the impact of genetic variation on MAO-A activity is scarce, but a few polymorphisms were described to modulate expression and catalytic activity (Hotamisligil and Breakefield, 1991; Jansson et al., 2005; Zhang et al., 2010). In particular, a MAO-A promoter variable number tandem repeat (VNTR) has been studied experimentally and in clinical association studies (Sabol et al., 1998; Deckert et al., 1999; Liu et al., 2013). However, the correlation between MAO-A genotype and phenotype is not fully clear (Cirulli and Goldstein, 2007), and, to the best of our knowledge, the relation with tyramine pharmacokinetics has not yet been studied in humans.

Systemic tyramine concentrations could also be influenced by other enzymes, such as CYP2D6. The genetically highly polymorphic CYP2D6 converts tyramine to dopamine (Hiroi et al., 1998; Bromek et al., 2011), and it was proposed that CYP2D6 could be involved in the hepatic elimination of tyramine (Niwa et al., 2011). However, it has not yet been studied in humans whether tyramine pharmacokinetics depend on the CYP2D6 genotype.

At physiological pH, 99.9% of tyramine is protonated and is thus unable to readily enter cells by passive diffusion. Earlier studies have reproducibly demonstrated that tyramine is a substrate of the organic cation transporter 1 (OCT1, or SLC22A1), and that frequent naturally occurring variants in OCT1 have reduced or absent activity in cellular uptake of tyramine (Breidert et al., 1998; Seitz et al., 2015). Thus, for hepatic clearance of systemically available tyramine in humans, OCT1 might play a relevant role, with reduced clearance in carriers of decreased or zero function OCT1 alleles. The OCT1 genotype could therefore be another possible factor that determines an individual’s response to tyramine.

Tyramine pharmacogenetics may also be of general biological interest, since the protection from potentially toxic substances could have been a driving force for the evolution of genetically polymorphic drug membrane transporters and drug metabolising enzymes.

The aim of the present study was to elucidate the genetic factors that determine systemic tyramine exposure and tyramine blood pressure effects. To this end, the pharmacokinetics of tyramine were studied in 88 healthy volunteers after oral administration of 400 mg of tyramine. Furthermore, the impact of MAO-A, CYP2D6, and OCT1 genotypes on the pharmacokinetics of tyramine and its main metabolite 4-HPAA as well as on tyramine blood pressure effects was analysed.

## Materials and Methods

### Subjects and Study Design

In this open-label pharmacokinetic/pharmacodynamic study, pharmacokinetics and effects of 400 mg oral tyramine were analysed in relation to MAO-A, CYP2D6, and OCT1 genotypes in 88 healthy volunteers. Eighty percent of the participants were unselected volunteers from Göttingen area in Germany; the remaining 20% were selected from an internal database to enrich the study sample with rare OCT1 or CYP2D6 variants. With widely accepted statistical criteria (type I error of 0.05 and type II error of 0.2), at least 10 subjects with genetically defined very low activity of OCT1 or CYP2D6 and a similar or higher number without deficiency should be sufficient to confirm a relevant 50% change in tyramine plasma AUC as a result of genetic variation. Because of the very diverse and not pharmacokinetic-based literature data on the functional effects of MAO-A polymorphisms, no power calculation could be performed with regard to MAO-A genotypes. However, a sample size of nearly a hundred individuals should be sufficient to find genotypes with a population frequency of at least 5%. All volunteers gave their written informed consent before participation in the study. The study was approved by the ethics committee of the University of Göttingen. It was registered at the German Clinical Trials Registry and the World Health Organization International Clinical Trials Registry Platform (DRKS00008915; https://www.drks.de/DRKS00008915).

Healthy male and female volunteers aged between 18 and 50 years with a body mass between 50 and 100 kg and a body mass index of 18–30 were eligible for inclusion. Volunteers with known intolerance to tyramine or those who required regular drug treatments other than oral contraceptives were not included. Further reasons for exclusion were any chronic illnesses that significantly raise the risk for cardiovascular events. All subjects were healthy according to a detailed medical history, medical examination, electrocardiogram, urine status and clinical chemistry, and haematology parameters (sodium, potassium, total bilirubin, aspartate aminotransferase, alanine-aminotransferase, creatinine, C-reactive protein, thyroid stimulating hormone, haemoglobin, erythrocyte, thrombocyte, and leucocyte counts).

Tyramine (99% purity; Sigma-Aldrich, Darmstadt, Germany) was recrystallised from double-distilled water and stored at room temperature. Purity was confirmed by HPLC. Gelatine capsules containing 400 mg of tyramine were prepared and stored at room temperature. To avoid interference by dietary tyramine, food and drink (including alcohol) consumption was prohibited from the evening before the study day until the last blood and urine sampling, except for water at any time and bread with butter 3 h before and 3 h after tyramine administration. A medical examination and electrocardiogram measurement were conducted 3 h prior to tyramine administration. Blood samples were drawn 3 h before and at the following times after tyramine intake: 15, 30, and 45 min as well as 1, 1.5, 2, 2.5, 3, 4, and 6 h. Urine samples were taken from 3 h to 10 min before tyramine administration as well as from 0 to 3 h and from 3 to 6 h after. Blood samples were centrifuged (10 min, 4°C, 4,000 rpm) and plasma and urine samples were stored at −20°C until analysis. Blood pressure and heart rate were measured 3 h, 35 min, and 10 min before tyramine administration, and subsequently in 15 min intervals until 6 h after. In a subgroup of eight participants, blood pressure was continuously monitored based on an analysis of pulse pressure curves using the SOMNOtouch^™^ NIPB non-invase system (SOMNOmedics GmbH, Randersacker, Germany). An orthostatic blood pressure test was performed 10 min before as well as 30 min and 1.5 h after tyramine intake. Pupillometry was performed 35 min prior to, and 1, 2, and 6 h after tyramine administration. Participants were asked to report on symptoms possibly related to tyramine (headache, fatigue, sleepiness, visual or hearing impairments, restlessness, nausea, dizziness, dry mouth, tremor, sensation of cold, and heart palpitation) using visual analogue scales 3 h before and 1, 3, and 6 h after tyramine intake.

### Bioanalytics

Plasma and urine concentrations of tyramine and 4-HPAA were analysed by HPLC with tandem mass spectrometry using tyramine-d4 (Santa Cruz Biotechnology, Inc., Dallas, TX, USA) as internal standard. GR 49336 (5-[[(methylamino)sulfonyl]methyl]-1*H*-indole-3-acetic acid; Santa Cruz Biotechnology, Inc.) was used as internal standard for 4-HPAA. The analytical standards for tyramine were obtained from Fluka (Morristown, NJ, USA) and for 4-HPAA from Sigma-Aldrich (Darmstadt, Germany). The limit of detection for tyramine was 0.1 ng/ml in plasma and 10 ng/ml in urine; for 4-HPAA, it was 5 ng/ml in plasma and 0.5 µg/ml in urine. The limit of quantification for tyramine was 0.25 ng/ml in plasma and 15 ng/ml in urine; for 4-HPAA, it was 15 ng/ml in plasma and 1.5 µg/ml in urine. Plasma and urine concentrations of dopamine and *N*-methyltyramine were found to be below the limit of detection of 1 ng/ml.

200 µl of plasma were precipitated in 1.5 ml Eppendorf tubes with 400 µl acetonitrile/methanol (9:1, v:v), including the internal standards. After 10 min on ice, tubes were centrifuged for 30 min at 13,000 rpm, and 300 µl of the supernatant were transferred to a microtiter plate. The supernatant was evaporated using nitrogen and re-dissolved in 0.1% formic acid. 10 µl were injected into the HPLC system. Chromatography was performed using a Shimadzu Nexera system with an API 4000 detector. Separation for analysis of tyramine and methyltyramine was performed at 40°C on a Brownlee SPP RP-Amide 100 x 4.6 mm, 2.7 µm particle size, column using water with 0.1% (v/v) formic acid, 2.57% acetonitrile, and 0.43% methanol as eluent. Detection (positive mode) of tyramine was based on the masses 139.1/121.1 and for tyramine-d4 on 142.0/125.0. Calibration range was 0.5 to 200 µg/l with coefficients of variation of 4.6%, 2.8%, and 2.6% at 2, 20, and 150 µg/l, respectively. Analysis of 4-HPAA was performed on the same system, using 0.1% formic acid, 17.14% acetonitrile, and 2.86% methanol as eluent and detection in the negative mode based on the masses 151.0/107.2 for 4-HPAA and 281.2/142.1 for the internal standard. Calibration range was between 0.03 and 20 mg/l and coefficients of variation were 4.7%, 4.2%, and 3.7% at 1, 5, and 15 mg/l.

### Genotyping

Genomic DNA was isolated from venous blood samples *via* automated solid phase extraction (EZ1 DNA Blood kit; Qiagen, Hilden, Germany). Genotyping was performed using single-base primer extension, as described previously (Seitz et al., 2015). The following genetic polymorphisms were analysed for MAO-A: rs2064070, rs6323, rs1137070, rs909525, rs2072743, rs1800464, rs1799835, and the 30-bp-VNTR in the proximal promoter region (Sabol et al., 1998); for CYP2D6: *2, *3, *4, *5, *6, *9, *10, *35, *41, and gene duplication; for OCT1: S14F (rs34447885), S189L (rs34104736), R61C (rs12208357), Q97K (c.289C > A), P117L (rs200684404), R206C (c.616C > T), G401S (rs34130495), M420del (rs72552763), G465R (rs34059508), C88R (rs55918055), and G220V (rs36103319).

### Statistics

The primary endpoint was the AUC of tyramine in plasma. Secondary endpoints were the other pharmacokinetic parameters of tyramine and its metabolite 4-HPAA, as well as heart rate, blood pressure, pupil size effects, and possible adverse events (headache, fatigue, visual or hearing impairments, restlessness, nausea, dizziness, dry mouth, tremor, hypothermia, heart palpitations). In addition, the cumulative amounts of tyramine and 4-HPAA excreted in urine were measured over time periods of 3 h before (baseline) and 6 h after application.

Pharmacokinetic parameters were calculated by non-compartmental analysis using Phoenix 64 WinNonlin version 6 (Certara USA, Inc., Princeton NJ, USA). AUC_inf_ of tyramine was calculated from time of dosing using the linear/log trapezoidal rule, and extrapolated to inﬁnity based on the last predicted concentration and using the terminal elimination rate constant (lambda z). Further parameters included the total plasma clearance after oral administration (CL/F) and the terminal half-life (t_1/2_), which were calculated as CL/F = dose/AUC_inf_ and t_1/2_ = ln(2)/lambda z.

The correlation between tyramine plasma AUC (primary endpoint) and MAO-A, CYP2D6, and OCT1 genotype was calculated *via* multiple regression analysis. The genotypes were categorised into 0, 1, or 2 active alleles for OCT1 and into 0, 0.5, 1, 1.5, 2, or >2 active alleles for CYP2D6, depending on their known effects on transporter/enzyme activity.

## Results

In total, 88 healthy volunteers (54 female, 34 male) participated in the study. The subjects were between 18 and 49 years of age (**Table 1**). The participants were genotyped for various well-known and common MAO-A, CYP2D6, and OCT1 polymorphisms (further data in **Supplementary Table 1**). All participants received 400 mg tyramine orally. There were no serious adverse events caused by tyramine.

**Table 1:** Characteristics of the study population (n = 88).

	Mean	Median	SD	Minimum	Maximum
**Age**	27.0	25	6.7	18	49
**Body height (cm)**	174.0	174.5	8.8	157	199
**Body mass (kg)**	69.9	68.7	11.2	49.0	99.7
**Body mass index**	23.0	22.7	2.77	18.4	29.8
	**n (%)**				
**Sex**	34 (39%) male				
**Smoking habit**	21 (24%) smokers				
**Alcohol drinking habit**	82 (93%) consumers				
**Oral contraceptive use** (females only)	27 (50%) yes				

### Pharmacokinetics of Tyramine and 4-HPAA

Baseline plasma concentrations of tyramine were below the limit of quantification in 98% of participants; in the remaining two subjects, the mean (individual values) concentration was 0.39 (0.25 & 0.53) ng/ml. The corresponding baseline plasma 4-HPAA concentrations were less than the limit of quantification in 84%, and mean (range) concentration in the remaining subjects was 70 (31–201) ng/ml. Baseline tyramine recovery in the urine cumulated for 3 h prior to tyramine administration was lower than the limit of quantification in 2% of participants; for the remaining volunteers, the mean (range) was 0.055 (0.007–0.222) mg. The baseline urinary recovery of 4-HPAA was below the limit of quantification in 26%, and mean (range) in the remaining subjects was 2.65 (0.67–9.05) mg.

**Figure 2:**
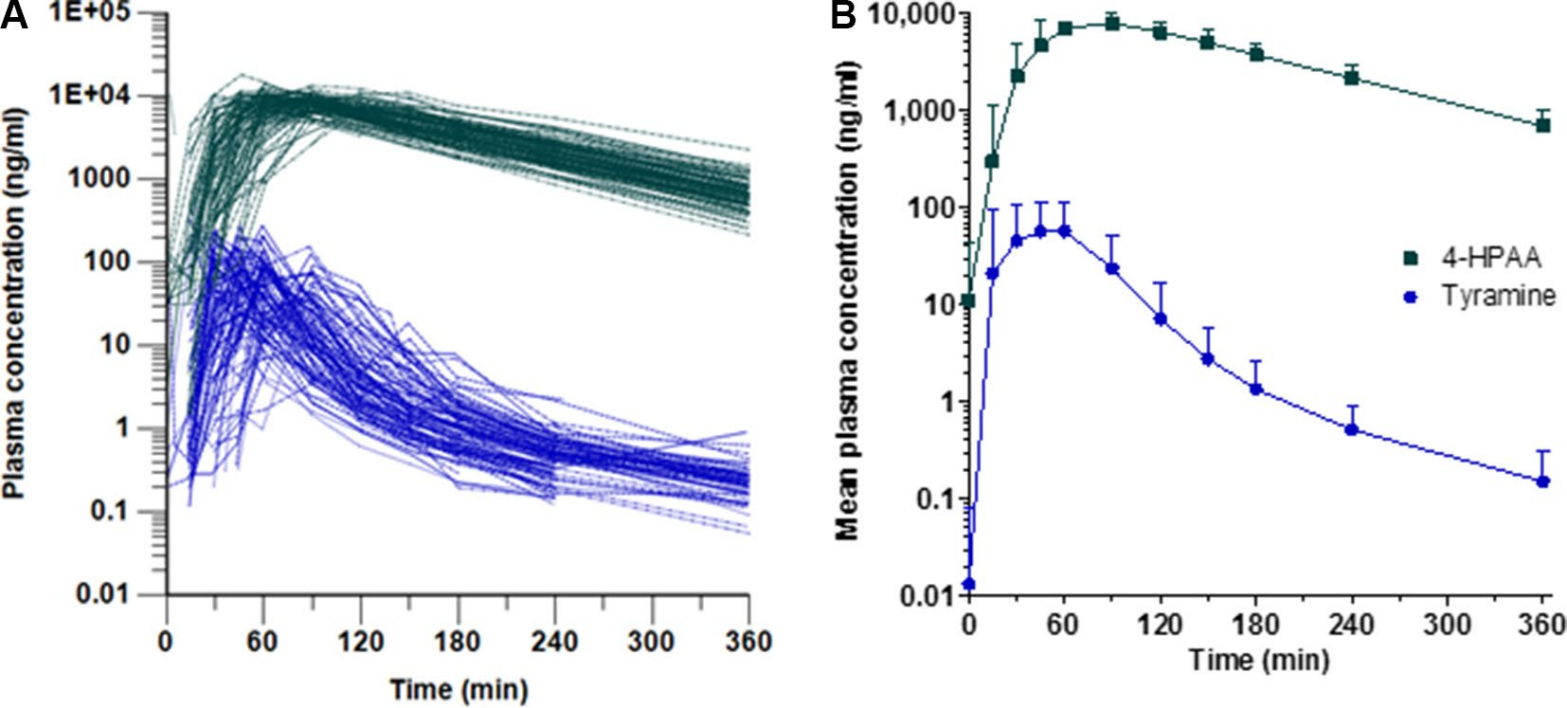
Plasma concentrations of tyramine (blue circles) and 4-HPAA (green squares) over time, shown **(A)** for each participant and **(B)** as means + SD (n = 88).

**Figure 3:**
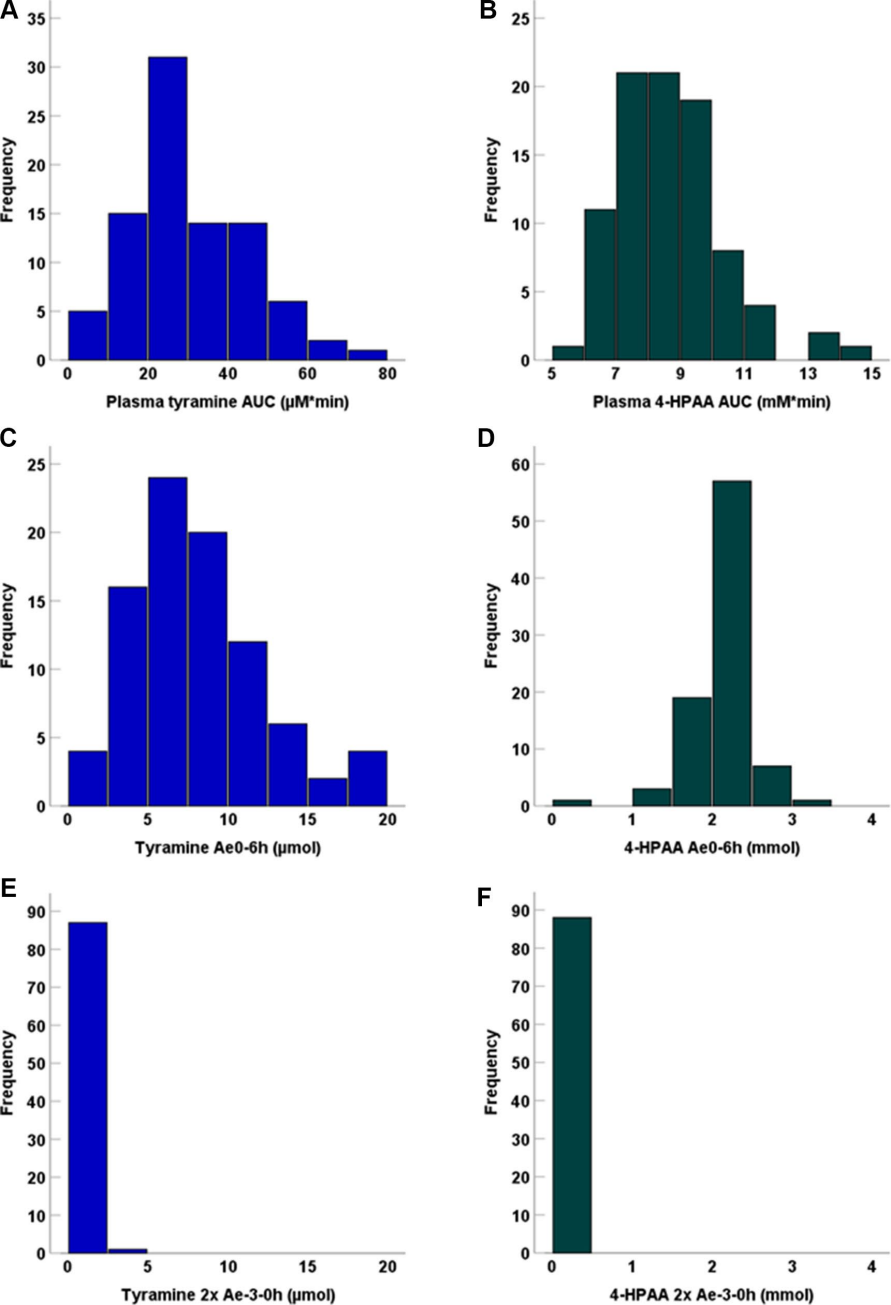
Frequency distribution of plasma AUCs for **(A)** tyramine and **(B)** 4-HPAA, as well as **(C)** tyramine Ae_0-6h_, **(D)** 4-HPAA Ae_0-6h_, **(E)** tyramine Ae_-3-0h_, and **(F)** 4-HPAA Ae_-3-0h_. Baseline Ae (Ae_-3-0h_) data were multiplied by 2 for better comparison to the 6h post-application measurement interval.

Following tyramine administration, increases in the plasma concentrations of tyramine and the metabolite 4-HPAA were seen in all participants. The mean plasma 4-HPAA concentrations were at any time point 14- to 4,600-fold higher than those for tyramine (**Figure 2**). In spite of highly standardised conditions for dosing and intake, t_max_ of tyramine was very diverse, ranging between 13 and 120 min. A large variation in t_max_ was also observed for 4-HPAA, which ranged from 29 to 150 min (**Table 2**). Tyramine and 4-HPAA t_max_ correlated strongly (p < 0.001). The AUCs (primary endpoint) and the amounts excreted during the first 6 h following tyramine administration (Ae_0-6h_) varied strongly for tyramine but were more uniform for 4-HPAA (**Figure 3**). In the 0–6 h urine fractions, the median of the dose recovered as the sum of tyramine and 4-HPAA was 72.2%, of which 99.8% was in the form of the metabolite. Given the high clearance to bioavailability ratio of 107 l/min, the bioavailability of tyramine is, as known, very low. It is most likely less than 1% when assuming that the total systemic clearance is not higher than 1 l/min. The higher plasma concentrations of 4-HPAA and the relatively high urinary recovery in form of the metabolite indicates that tyramine is most likely absorbed at the intestinal mucosa and directly metabolised there by MAO-A, or immediately afterwards in the liver, before reaching the systemic circulation. This intensive first-pass effect is, however, apparently not sufficient to prevent a significant increase in blood pressure in some of the study participants.

**Table 2:** Pharmacokinetic parameters of tyramine and 4-HPAA.

	Tyramine	4-HPAA
**AUC**_inf_ (min*µg/ml)	3.74 (1.31–10.7)	1298 (1249–1350)
**t**_1/2_ (min)	55 (25–124)	73 (71–75)
**t**_max_ (min)	46 (13–120)	88 (29–150)
**C**_max_ (µg/ml)	0.087 (0.019–0.398)	9.04 (8.66–9.44)
**CL/F** (l/min)	107 (38–305)	
**CL**_renal_ (l/min)	0.26 (0.11–0.61)	0.24 (0.09–0.60)
**Ae**_0-6h_ (mg)	0.98 (0.34–2.77)	307 (130–729)
**2x Ae**_-3-0h_ (mg)	0.089 (0.025–0.316)	4.32 (1.26–14.84)

Data is given as geometric mean with the geometric 95% confidence interval in parentheses, except for t_max_, for which the median and the range is given; n = 88. Baseline amount excreted (Ae_-3-0h_) data were multiplied by 2 for better comparison to the 6 h post-application measurement interval.

**Figure 4:**
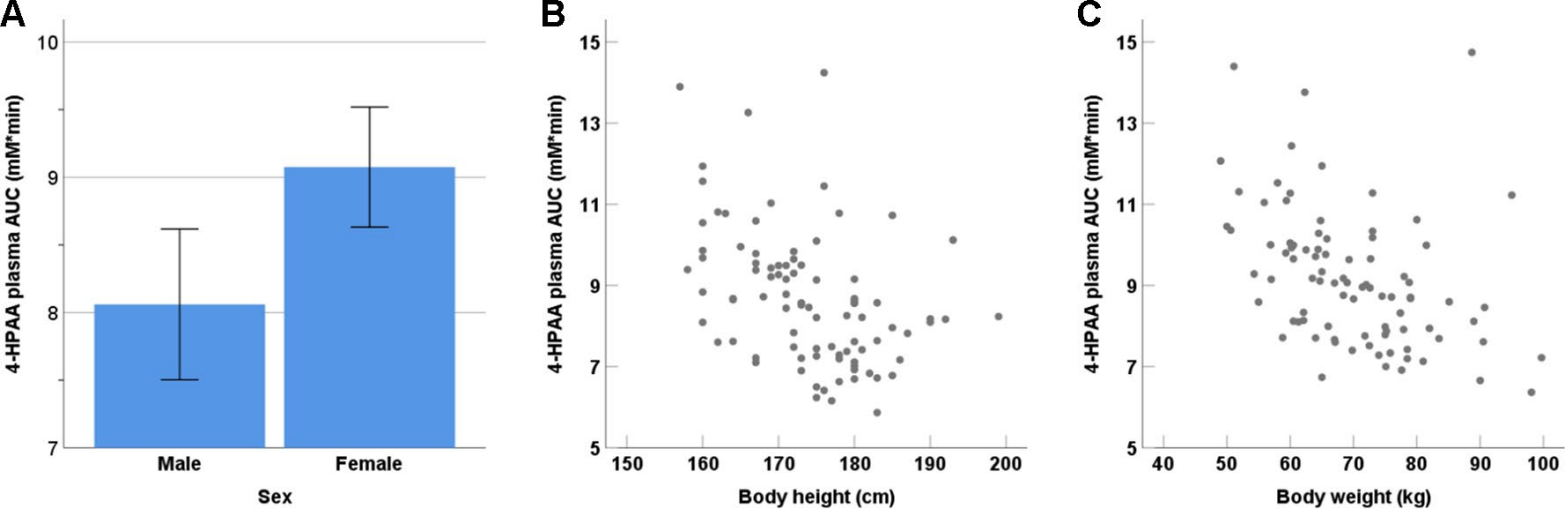
Dependence of the tyramine metabolite 4-HPAA on **(A)** sex (p = 0.001), **(B)** body height (p < 0.001; two-tailed), and **(C)** body weight (p < 0.001; two-tailed).

**Figure 5:**
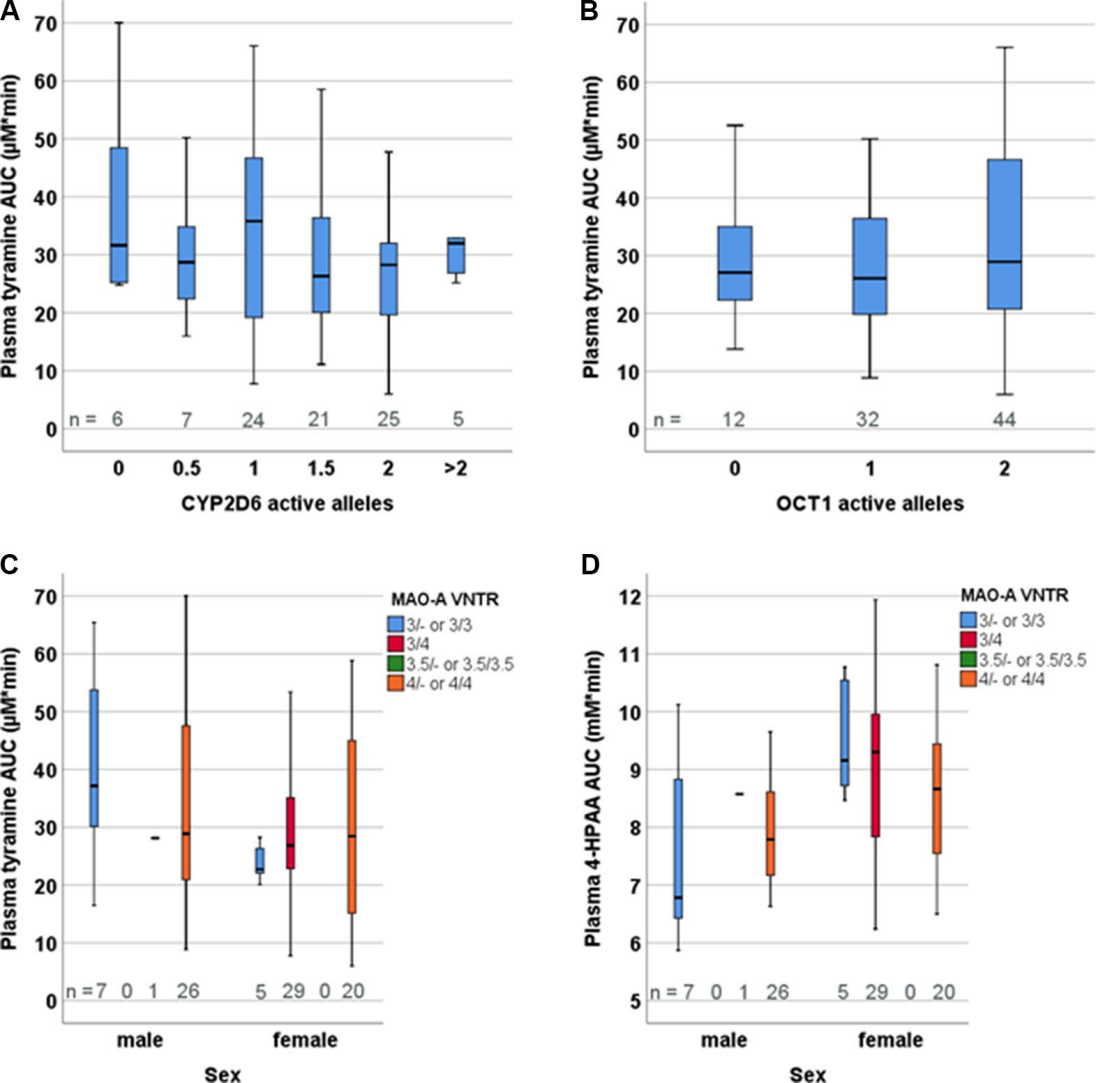
Distribution of tyramine plasma AUCs based on the genotypes of **(A)** CYP2D6, **(B)** OCT1, and **(C)** MAO-A VNTR. **(D)** Distribution of 4-HPAA plasma AUCs in relation to the MAO-A VNTR genotype.

We observed no statistically significant dependence of tyramine AUC on age, weight, height, body mass index, sex, smoking status, consumption of alcohol, or the use of oral contraceptives, but tyramine Ae_0-6h_ varied significantly by sex (male 9.69 µmol, female 7.07 µmol; p = 0.002, Mann-Whitney U test). A negative correlation was seen for 4-HPAA AUC with body height (p < 0.001, two-tailed) and body weight (p < 0.001, two-tailed, **Figure 4**). Furthermore, 4-HPAA plasma AUC was lower in male compared to the female participants (p = 0.005, two-tailed t-test; **Figures 4** and **5**).

### Pharmacogenetics of Tyramine and 4-HPAA

The participants were genotyped for the polymorphisms in the MAO-A, CYP2D6, and OCT1 genes that are presumed or known to be relevant in European populations. These include for MAO-A: rs2064070, rs6323, rs1137070, rs909525, rs2072743, rs1800464, rs1799835, and the 30-bp-VNTR in the proximal promoter region (Sabol et al., 1998). Since *MAOA* is found on the X-chromosome, males and females were analysed separately. None of the MAO-A polymorphisms presumed, according to the literature, to influence expression levels or result in amino acid exchange had any significant impact on tyramine or 4-HPAA pharmacokinetics, analysed as plasma AUC_inf_ (the pre-defined primary endpoint), Ae_0-6h_, and metabolic ratio calculated as the ratio of the AUCs of tyramine and 4-HPAA (**Figure 5**, **Supplementary Figure 1**, **Supplementary Table 1**).

All frequent CYP2D6 variants typically relevant in European populations (the alleles *2, *3, *4, *5, *6, *9, *10, *35, *41, and gene duplication) were analysed, but there was not any relation between tyramine or 4-HPAA pharmacokinetic parameters and the CYP2D6 genotype-based activity scores (**Figure 5**). Dopamine could nevertheless be formed from tyramine by CYP2D6 but only to a very minor extent. We therefore looked at physiological parameters potentially indicating CYP2D6-dependent dopamine formation, such as systolic and diastolic blood pressure or effects of dopamine in the central nervous system, including pupillometry. However, none of the parameters differed between the groups defined by the CYP2D6 genotype.

We hypothesised at the beginning of this study that the genetically highly polymorphic OCT1 could be involved in hepatic uptake of tyramine from the blood circulation, as tyramine is a substrate with nanomolar affinity (Breidert et al., 1998). Thus, 12 homozygous or compound heterozygous carriers of OCT1 alleles that are known to confer very low to absent tyramine transport activity *in vitro* were included (Seitz et al., 2015). Altogether, the following variants were investigated: S14F (rs34447885), S189L (rs34104736), R61C (rs12208357), Q97K (c.289C > A), P117L (rs200684404), R206C (c.616C > T), G401S (rs34130495), M420del (rs72552763), G465R (rs34059508), C88R (rs55918055), and G220V (rs36103319). However, neither the pharmacokinetic parameters (**Figure 5**) nor the blood pressure response or other parameters varied among the OCT1 genotypes. Furthermore, analyses differentiating between the specific OCT1 alleles did not show statistically significant differences (**Supplementary Table 1**).

### Effects on Blood Pressure and Heart Rate

**Figure 6:**
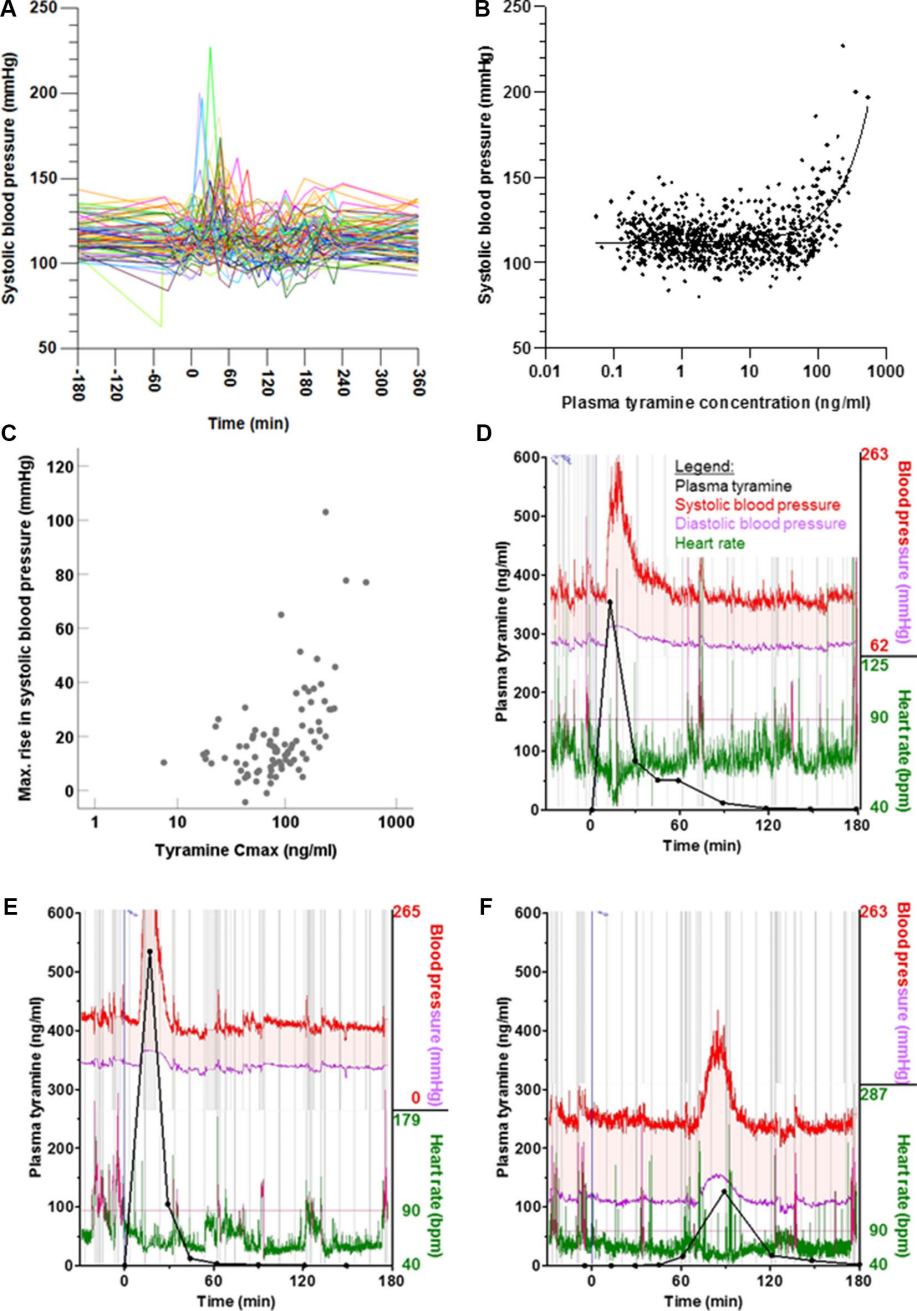
(A) Time course of systolic blood pressure shown for each participant. (B) Systolic blood pressure relative to plasma tyramine concentration, shown as individual readings. More information and similar representations for diastolic blood pressure and heart rate are shown in **Supplementary Figure 2**. (C) Correlation between maximum systemic tyramine concentration and maximum rise in systolic blood pressure (p < 0.001, two-tailed). (D–F) Individual exemplary time courses of blood pressure and heart rate, measured at very short intervals *via* the pulse transit time. For reference, the plasma concentrations of tyramine have been superimposed.

**Figure 7:**
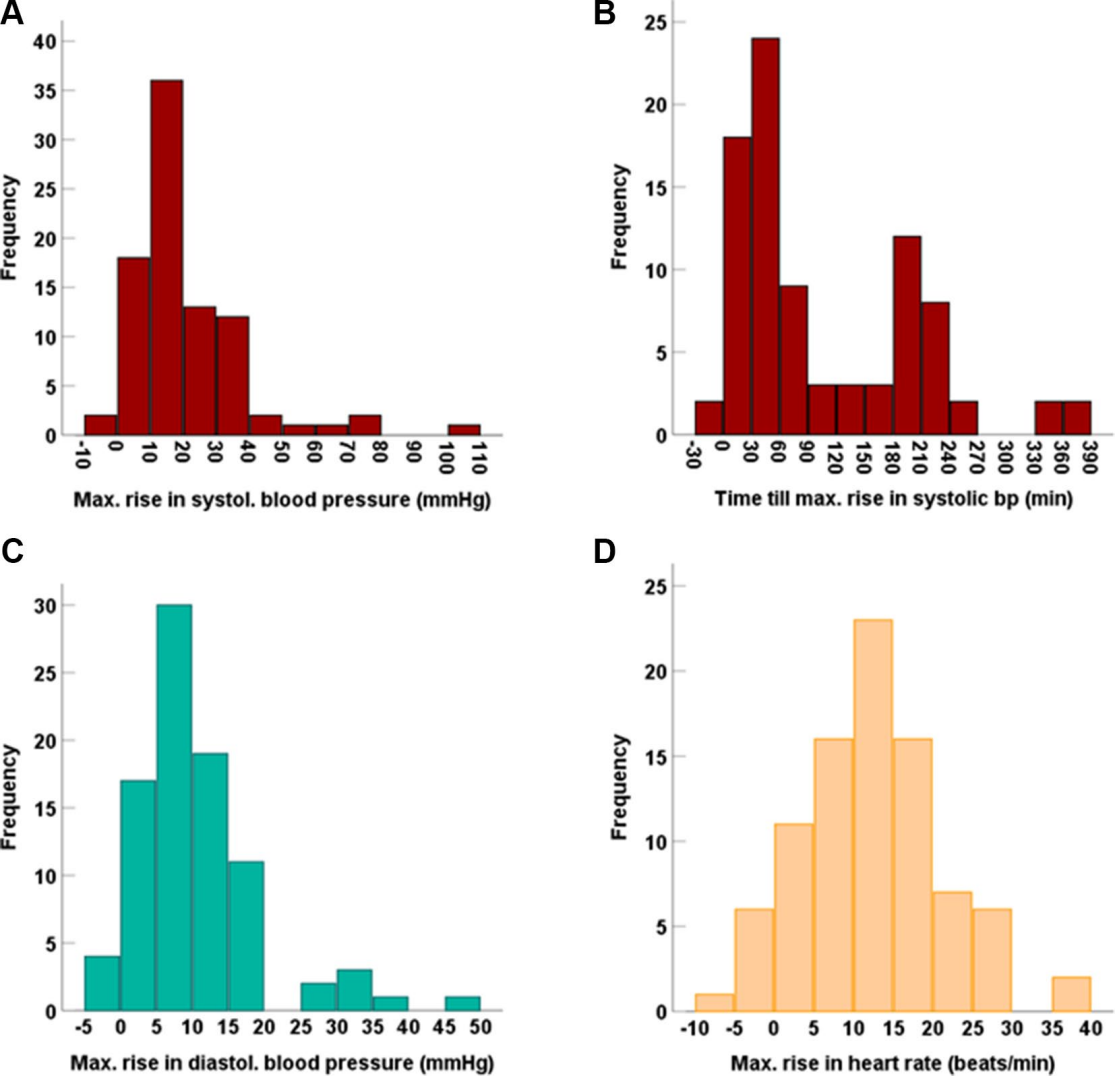
Frequency distribution of the maximum increases in **(A)** systolic blood pressure and **(B)** the time till this was reached, as well as of the maximum increases in **(C)** diastolic blood pressure and **(D)** heart rate.

Following tyramine administration, systolic blood pressure increased by more than 10 mmHg in 71% of the volunteers. Concentration-effect analyses showed a rise in systolic blood pressure at tyramine plasma concentrations of approx. 60 µg/l and above (**Figure 6**). The diastolic blood pressure increased at concentrations higher than 100 µg/l (**Supplementary Figure 2**). As with the systemic tyramine exposure, strong interindividual variations were seen in blood pressure response, and for the systolic blood pressure in particular (reaching increases in systolic blood pressure by up to 103 mmHg above baseline values measured on the same day; **Figure 7**). As illustrated, the maximum rise in systolic blood pressure correlated strongly with the maximum systemic tyramine concentrations.

Beside conventional sphygmomanometric blood pressure measurements at 15-min intervals, blood pressure was measured *via* the pulse transit time at every heart beat. It showed that the blood pressure peaks triggered by tyramine can be very strong but were usually short-lived. This is illustrated by the blood pressure reactions of three participants in **Figure 6**. They are not, however, representative for all volunteers, as the observed blood pressure effects varied greatly not only with respect to the intensity but also in the time of onset. For instance, in one participant (**Figure 6**), the blood pressure reaction occurred much delayed at 90 min after tyramine administration.

The interindividual variation could not be linked to the individual’s MAO-A, CYP2D6, or OCT1 genotypes; no difference in the maximum increases in systolic blood pressure, diastolic blood pressure, and heart rate was found for the polymorphisms investigated here (data not shown).

### Adverse Symptoms

**Figure 8:**
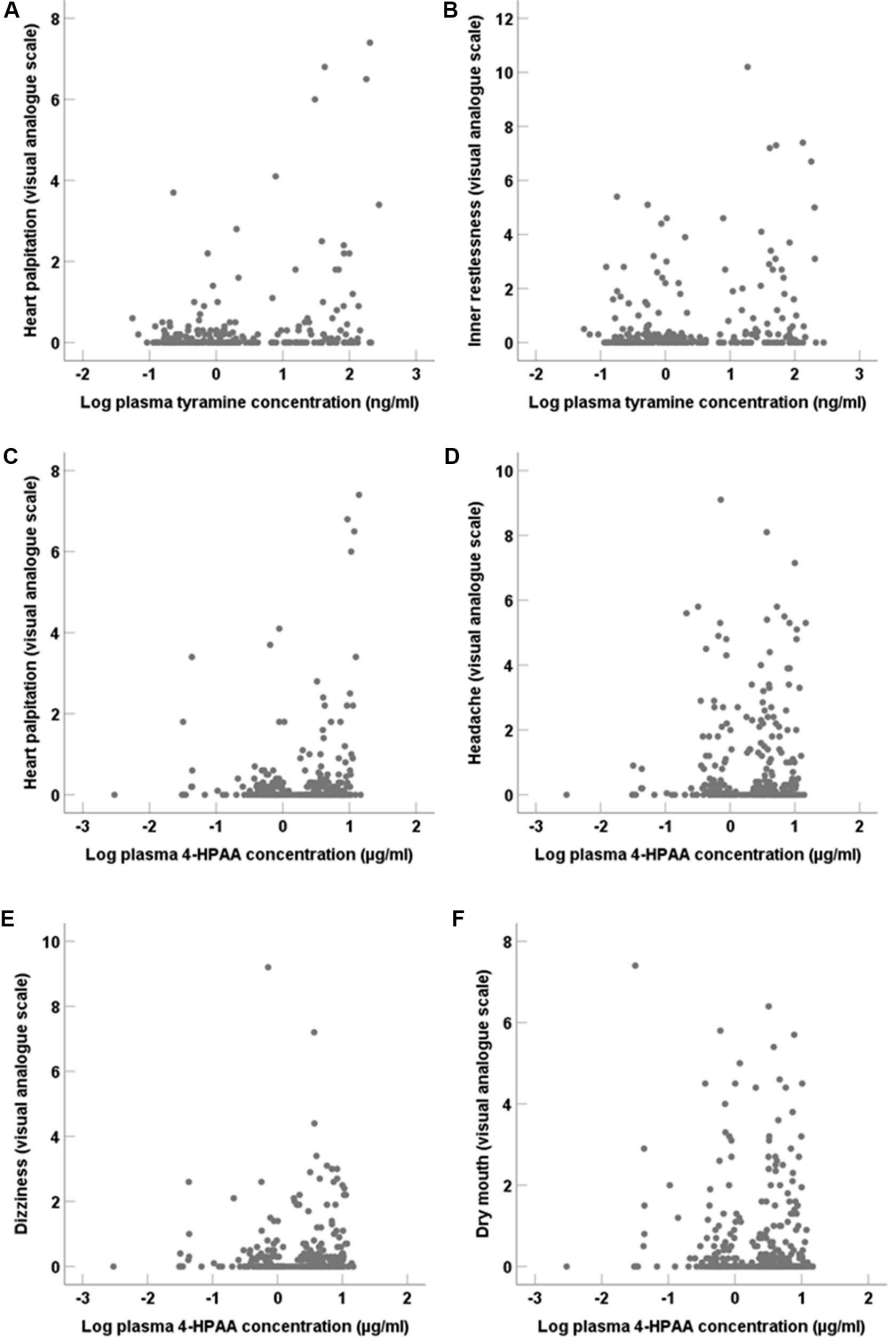
Adverse symptoms assessed by visual analogue scales in correlation (two-tailed) to systemic tyramine and 4-HPAA concentrations. **(A)** Heart palpitation (p < 0.001) and **(B)** Inner restlessness (p = 0.02) in correlation to tyramine. **(C)** Heart palpitation (p < 0.001), **(D)** headache (p = 0.01), **(E)** dizziness (p = 0.03), and **(F)** dry mouth (p = 0.049) in correlation to systemic 4-HPAA concentration.

Possible adverse symptoms of tyramine were assessed by visual analogue scales. Positive correlations (two-tailed; **Figure 8**) were seen for systemic tyramine concentration with heart palpitation (p < 0.001) and inner restlessness (p = 0.02). Systemic 4-HPAA concentration also correlated (two-tailed; **Figure 8**) positively with heart palpitation (p < 0.001) as well as with headache (p = 0.01), dizziness (p = 0.03), and weakly with dry mouth (p = 0.049). Repeated pupillometric analyses to detect possible central adrenergic effects by tyramine showed no difference in pupil size and the pupil reaction to light before and after tyramine administration (data not shown).

## Discussion

To summarise, our results indicate that OCT1 and CYP2D6 are of little or no importance for tyramine pharmacokinetics. In contrast, the study confirmed the relevance of MAO-A, but indicated that functionally relevant genomic variation in MAO-A is apparently not frequent. Clearly, our data show that pharmacokinetics of tyramine varies greatly between individuals. However, the genetic polymorphisms that account for this variation still remain elusive.

A likely candidate would have been MAO. As the pressor response is associated with the inhibition of MAO-A but not MAO-B (Finberg and Tenne, 1982; Finberg, 2014), only MAO-A should significantly influence systemic exposure to tyramine. There is extensive literature on the possible medical impact of inherited polymorphisms in MAO-A. Most of the more than 500 publications on potential effects of MAO-A polymorphisms correlated polymorphisms in or near the MAO genes with complex neuropsychological or psychiatric phenotypes (Naoi et al., 2016). Literature data that links MAO-A polymorphisms to the enzyme’s expression or catalytic activity is scarce, and to the best of our knowledge, no *in vivo* metabolic phenotypes have been analysed in relation to MAO-A polymorphisms. Those polymorphisms were selected for investigation for which an association had been described, such as the 30-bp-VNTR localised 1.2 kb upstream of the MAO-A transcription starting site. Regarding this VNTR, Deckert and colleagues showed in luciferase reporter gene assays that the transcriptional activity was approximately 1.7-fold higher for the 3.5-repeat- compared to the 3-repeat-variant (Deckert et al., 1999). However, it was rare (< 3%) in the German population sample and was found in only one participant of our study (**Supplementary Table 1**). The 4-repeat-variant was most abundant (61% prevalence in the German population sample) but was associated with merely 1.3-fold higher transcriptional activity compared to the 3-repeats-variant. Another study also observed 1.3-fold and 1.9-fold higher promoter activity for the 4-repeat- compared to the 3-repeat-allele in two different cell lines, while Sabol et al. found that the transcriptional efﬁciency was up to 4.6-fold and 4.8-fold higher in human cell lines carrying 3.5 or 4 repeats compared to those carrying 3 repeats (Sabol et al., 1998; Guo et al., 2008). For the single nucleotide polymorphism rs6323, a 3.8-fold higher mRNA expression and up to a 75% higher MAO-A activity was associated with the G-allele compared to the T-allele (Hotamisligil and Breakefield, 1991; Jansson et al., 2005; Leuchter et al., 2009; Zhang et al., 2010). With respect to rs1137070, a 2.3-fold higher mRNA expression was found in carriers of the T-allele in comparison to the C-allele, and for rs2072743, the mRNA expression was 3.8-fold higher with the A-allele compared to the G-allele (Zhang et al., 2010). The frequent polymorphisms rs2064070 and rs909525 were also included in our analysis. In addition, we were particularly interested in the functional effects of the MAO-A non-synonymous polymorphisms rs1800464 (R129W) and rs1799835 (V314F), but only one hemizygous male and three heterozygous female participants were carriers of the C-allele for rs1800464 and all participants had the same genotype with respect to rs1799835.

Despite some interesting indications from previous *in vitro* experiments, no effect was observed for any of these MAO-A polymorphisms on any relevant parameters reflecting tyramine pharmacokinetics or MOA-A-catalysed oxidative deamination resulting in 4-HPAA formation. It thus appears that genomic variation in MAO-A is not accountable for the variation in pharmacokinetics of tyramine. A possible explanation would be that the known genetic variants of MAO-A do not have a strong impact on the protein’s function *in vivo*, given the comparatively little (only up to 3-fold) variation in 4-HPAA plasma AUC and Ae_0-6h_ (**Figure 3**). No bimodal or trimodal frequency distributions indicative of monogenetic polymorphisms were seen (**Figure 3**). One may conclude that genotypes predicting phenotypes with very low or very high MAO-A activity in humans are rare. For most of the polymorphisms studied here, the expected effect would have been a change in gene expression, which typically depends on a number of transcription factors. As gene regulation may be different in the central nervous system, the absence of a strong effect of these polymorphisms on peripheral MAO-A may not exclude an effect on transcription in the brain.

Despite the lack of influence for MAO-A genotypes on tyramine biotransformation, it is unlikely that MAO-A is of little relevance to the pharmacokinetics of tyramine. This is because 76.8% of the applied tyramine dose was, on average, recovered in urine in form of 4-HPAA, the metabolite formed by MAO. Thus, our study confirmed in humans that MAO catalyses the main pathway in tyramine biotransformation and that tyramine might serve as a useful probe drug for studying MAO activity *in vivo*.

CYP2D6 is, due to its highly polymorphic nature, responsible for the variability in response to numerous substances, including more than 10% of all drugs. As was shown here, in a study with six deficient metabolisers and seven low activity intermediate metabolisers (the latter typically having less than 25% of usual activity) on the one side and four ultra-rapid metabolisers on the other side (**Supplementary Table 1**), there was no effect of CYP2D6 activity on tyramine pharmacokinetics or pharmacodynamics. Thus, CYP2D6 is irrelevant for the effects of dietary tyramine. Several studies found an association between CYP2D6 genotype and personality or brain imaging phenotypes (Llerena et al., 1993; Stingl et al., 2013). The formation of dopamine from endogenous precursors might be one mechanism behind these associations, but the present study does not support this hypothesis.

As more than 99.9% of tyramine is protonated at typical physiological pH in the relevant body compartments, tyramine should not be able to readily diffuse across membranes. Thus, its pharmacokinetics likely depend on a transport protein. In many subjects, tyramine was quite rapidly absorbed into the human body and appeared in the blood already 10 min after ingestion (**Figure 6**). The food interaction described for tyramine (VanDenBerg et al., 2003) may simply be due to slower gastric emptying with food, or, alternatively, may indicate competitive inhibition at transport proteins in the intestinal mucosa. OCT1 is highly expressed in liver sinusoidal membranes and only poorly expressed in the intestine. OCT1 genotypes should therefore be relevant for the hepatic clearance of tyramine from the systemic circulation rather than for intestinal absorption. However, our data provides no indication for that. Since the relevant hepatic uptake transporter for tyramine does not appear to be OCT1, it remains to be determined which other transporters could be responsible. Most tyramine was indeed recovered in urine in form of its metabolite 4-HPAA and thus had apparently entered the enterocytes and the hepatocytes. Other transporters that may participate in the absorption of organic cations in the small intestine include PMAT (*SLC29A4*), OCT3 (*SLC22A3*), OCTN1 (*SLC22A4*), OCTN2 (*SLC22A5*), NET1 (*SLC6A2*), DAT1 (*SLC6A3*), SERT (*SLC6A4*), CHT1 (*SLC5A7*), CTL1-5 (*SLC44A1*-*5*), and THRT2 (*SLC19A3*) (Zhou et al., 2007; Koepsell, 2015). However, their expression in the intestine may be relatively low and/or polymorphisms that strongly affect their function are not frequently found.

Tyramine was administered orally to resemble dietary tyramine, but the administered dose was higher than typical tyramine intake through nutrition. For instance, the tyramine concentration was found to be below 50 mg/kg for many different types of cheese, but can reach up to 1,000 mg/kg in matured cheese (Gillman, 2018). Other fermented foods, such as sausages, marmite, soybean products, and fish sauce, can also contain several hundred mg/kg of tyramine. Most wines and beers have concentrations of less than 10 mg/l, but a few were found to contain up to 100 mg/l. Fresh food products contain lesser amounts of tyramine, especially with modern production techniques and adequate food hygiene (Gillman, 2018). According to literature data, the pressor response occurs at oral tyramine doses in the fasting state of 200 mg upwards (VanDenBerg et al., 2003; Di Stefano et al., 2013; Gillman, 2018). Higher systemic tyramine concentrations (e.g. in combination with MAO-A inhibitors) would likely show stronger effects, but in light of the fact that, already in this study, increases in systolic blood pressure by up to 100 mmHg were observed in some participants, the risk for adverse effects would be too high. It remains to be clarified why high blood pressure peaks were only found in some individuals, whereas in 90% of the participants, increases in systolic blood pressure were less than 40 mmHg. Given the high concentration-effect relationship (**Figure 6**), this may not necessarily be a result of genomic variation but could be due to other effects, such as the speed of gastric emptying or the passage time through the small intestine.

To summarise, extensive interindividual differences in tyramine AUC and large variation in blood pressure effects that correlated strongly with plasma tyramine concentration were seen in 88 healthy volunteers following administration of 400 mg tyramine. Despite several types of evidence from *in vitro* data that inherited MAO-A, CYP2D6, or OCT1 polymorphisms could be the underlying reason for the variation, we did not observe such an association *in vivo*. The data on the urinary recovery of 4-HPAA, generated *via* MAO-A-catalysed oxidation, did not indicate the existence of common deficient, very low, or ultra-active MAO-A phenotypes in humans. This study confirms that the therapeutic significance of any experimental observation and its relevance for humans should be confirmed in clinical studies and cannot be inferred from *in vitro* data alone, no matter how well obtained.

## Data Availability Statement

The datasets generated for this study are available on request to the corresponding author.

## Ethics Statement

The studies involving human participants were reviewed and approved by the ethics committee of the University of Göttingen. The patients/participants provided their written informed consent to participate in this study.

## Author Contributions

MR and JB wrote the manuscript. FF, JB, SV, and JM designed the research. FF, JM, TP, MR, OJ, AG, FB, JB, RGB, UK, and MT performed the research. MR and JB analysed the data.

## Conflict of Interest

The authors declare that the research was conducted in the absence of any commercial or financial relationships that could be construed as a potential conflict of interest.

## Abbreviations

4-HPAA, 4-hydroxyphenylacetic acid; MAO, monoamine oxidase; OCT, organic cation transporter; VNTR, variable number tandem repeat.
