# Supplementary material for: Highly Variable Pharmacokinetics of Tyramine in Humans and Polymorphisms in OCT1, CYP2D6, and MAO-A
Source: Front Pharmacol. 2019 Oct 30;10:1297. doi: 10.3389/fphar.2019.01297 (PMC6831736; doi:10.3389/fphar.2019.01297)
Supplement: Supplementary file 1 [file DataSheet_1.pdf]

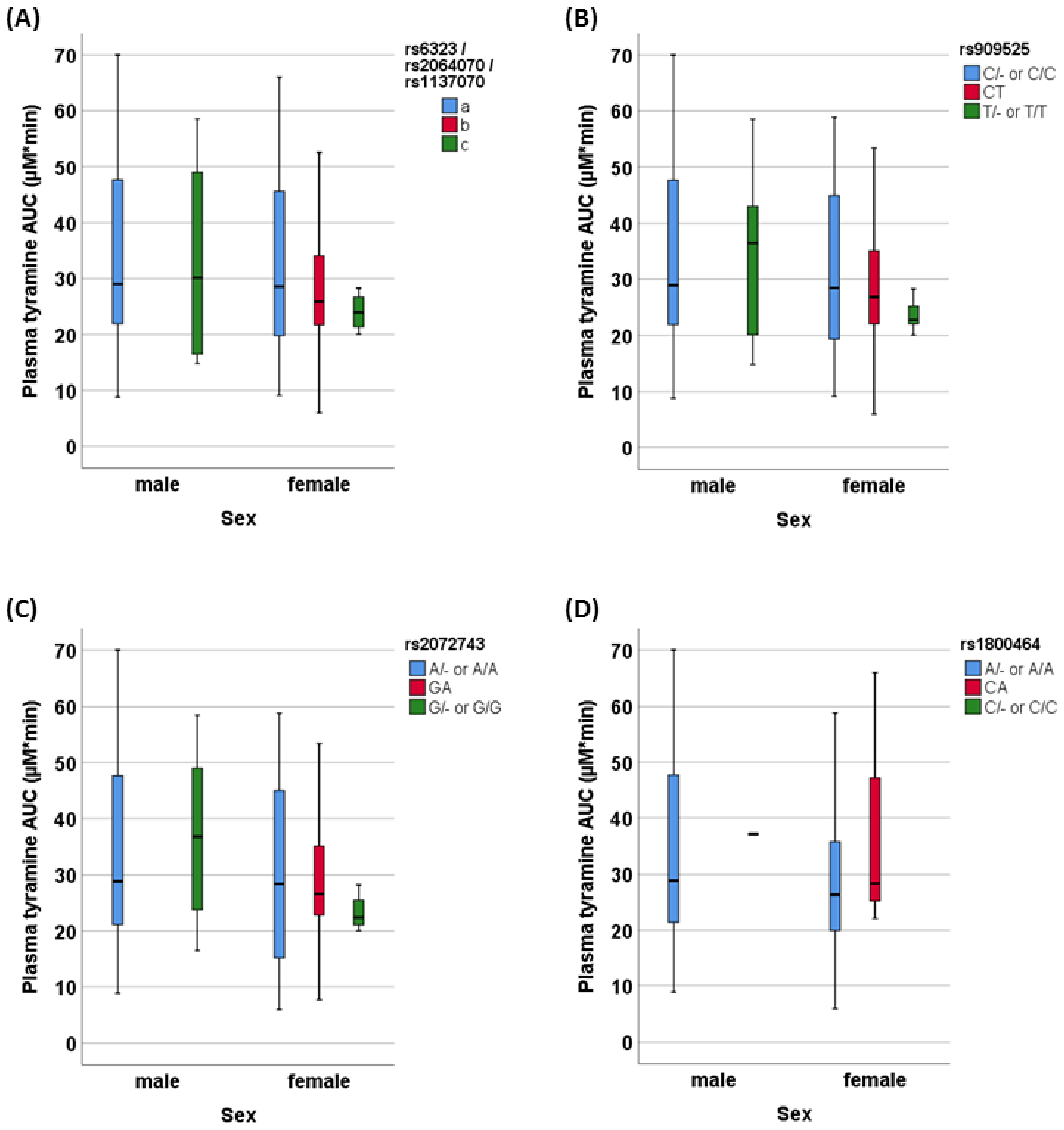

Supplementary Figure 1 Distribution of tyramine plasma AUCs in relation to the following MAO-A single nucleotide polymorphisms (SNP): **(A)** rs6323, rs2064070, and rs1137070. The diagrams for these three SNPs were identical, thus only one is shown here that represents all three SNPs. The variants are as follows: a, T/- or T/T for rs6323 and rs2064070 or C/- or C/C for 1137070; b, T/G for rs6323, T/A for rs2064070, or C/T for 1137070; c, G/- or G/G for rs6323, A/- or A/A for rs2064070, or T/- or T/T for 1137070 **(B)** rs909525, **(C)** rs2072743, **(D)** rs1800464. With respect to rs1799835, all 88 participants were carriers of the A/A-genotype

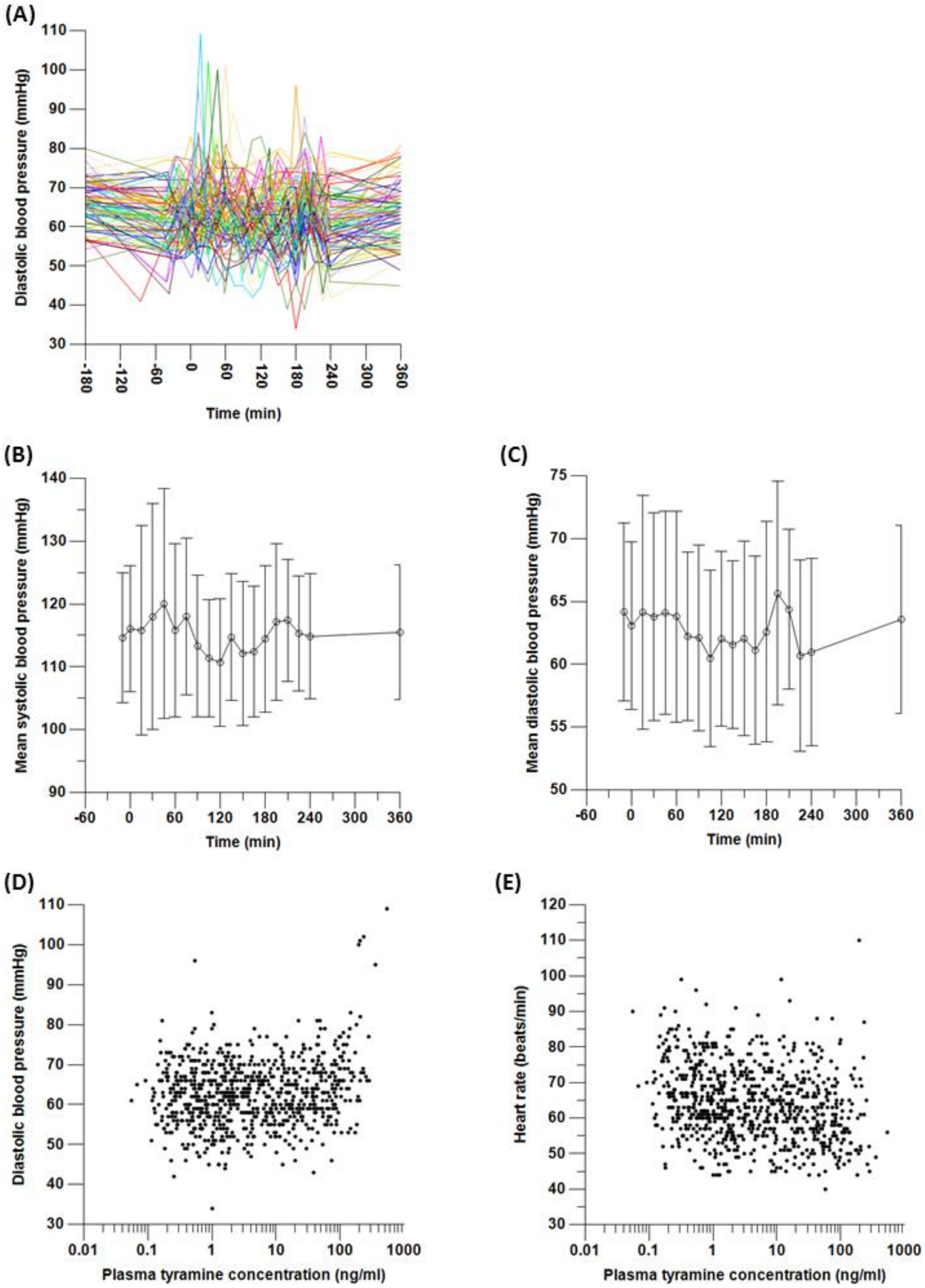

Supplementary Figure 2 (A) Time course of diastolic blood pressure, shown for each participant. **(B)** Systolic and **(C)** diastolic blood pressure, shown as means ± SD (n = 88). **(D)** Diastolic blood pressure and **(E)** heart rate relative to plasma tyramine concentration, shown as individual readings
