# Supplementary material for: Highly Variable Pharmacokinetics of Tyramine in Humans and Polymorphisms in OCT1, CYP2D6, and MAO-A
Source: Front Pharmacol. 2019 Oct 30;10:1297. doi: 10.3389/fphar.2019.01297 (PMC6831736; doi:10.3389/fphar.2019.01297)
Supplement: Supplementary file 2 [file Table_1.docx]

**Supplementary Table 1** Mean plasma AUC_inf_ and Ae_0-6h_ for tyramine and 4-HPAA relative to the MAO-A (male and female participants separately), CYP2D6, and OCT1 genotypes investigated in this study

| MAO-A poly-morphism | Genotype | n | Tyramine (males only) | | | | 4-HPAA (males only) | | | |
| --- | --- | --- | --- | --- | --- | --- | --- | --- | --- | --- |
|  |  |  | **AUC_inf_** (µM*min) | **SD** | **Ae_0-6h_**  (µmol) | **SD** | **AUC_inf_** (mM*min) | **SD** | **Ae_0-6h_** (mmol) | **SD** |
| VNTR | 3/- | 7 | 41.0 | 17.8 | 11.9 | 5.1 | 7.61 | 1.73 | 2.04 | 0.26 |
|  | 3.5/- | 1 | 28.1 |  | 10.0 |  | 8.57 |  | 1.86 |  |
|  | 4/- | 26 | 32.2 | 15.3 | 9.10 | 3.94 | 8.16 | 1.60 | 2.19 | 0.18 |
| rs2064070 | T/- | 28 | 34.0 | 15.6 | 9.42 | 3.92 | 8.07 | 1.63 | 2.16 | 0.20 |
|  | A/- | 6 | 33.2 | 17.9 | 10.9 | 5.6 | 8.01 | 1.58 | 2.09 | 0.26 |
| rs6323 | T/- | 28 | 34.0 | 15.6 | 9.42 | 3.92 | 8.07 | 1.63 | 2.16 | 0.20 |
|  | G/- | 6 | 33.2 | 17.9 | 10.9 | 5.6 | 8.02 | 1.58 | 2.09 | 0.26 |
| rs1137070 | C/- | 28 | 34.0 | 15.6 | 9.42 | 3.92 | 8.07 | 1.63 | 2.16 | 0.20 |
|  | T/- | 6 | 33.2 | 17.9 | 10.9 | 5.6 | 8.02 | 1.58 | 2.09 | 0.26 |
| rs909525 | C/- | 27 | 33.9 | 15.9 | 9.43 | 4.00 | 8.14 | 1.61 | 2.17 | 0.19 |
|  | T/- | 7 | 33.8 | 16.4 | 10.7 | 5.2 | 7.75 | 1.60 | 2.06 | 0.25 |
| rs2072743 | A/- | 28 | 33.2 | 16.0 | 9.34 | 3.95 | 8.09 | 1.60 | 2.16 | 0.19 |
|  | G/- | 6 | 36.9 | 15.5 | 11.3 | 5.3 | 7.90 | 1.70 | 2.07 | 0.27 |
| rs1800464 | A/- | 33 | 33.8 | 16.0 | 9.71 | 4.27 | 8.12 | 1.58 | 2.16 | 0.20 |
|  | C/- | 1 | 37.2 |  | 9.15 |  | 6.16 |  | 1.89 |  |
| rs1799835 | A/- | 34 | 33.9 | 15.8 | 9.69 | 4.20 | 8.06 | 1.60 | 2.15 | 0.21 |
|  | C/- | 0 |  |  |  |  |  |  |  |  |
| CA-repeat | 16/- | 1 | 70.0 |  | 11.1 |  | 8.24 |  | 1.81 |  |
|  | 17/- | 3 | 23.9 | 7.9 | 8.44 | 2.86 | 8.07 | 0.55 | 2.21 | 0.32 |
|  | 18/- | 20 | 30.4 | 11.8 | 9.00 | 4.00 | 8.12 | 1.71 | 2.18 | 0.17 |
|  | 19/- | 4 | 40.0 | 23.8 | 9.98 | 4.72 | 6.79 | 1.00 | 2.03 | 0.19 |
|  | 20/- | 1 | 57.0 |  | 14.3 |  | 10.8 |  | 2.37 |  |
|  | 22/- | 1 | 49.0 |  | 15.3 |  | 9.84 |  | 2.07 |  |
|  | 23/- | 1 | 23.8 |  | 6.09 |  | 10.1 |  | 1.69 |  |
|  | 24/- | 2 | 26.5 | 14.1 | 9.51 | 6.04 | 7.26 | 0.80 | 2.22 | 0.27 |
|  | 25/- | 1 | 58.5 |  | 18.5 |  | 6.78 |  | 2.34 |  |
| Total | Males only | 34 | 33.9 | 15.8 | 9.69 | 4.20 | 8.06 | 1.60 | 2.15 | 0.21 |

| MAO-A poly-morphism | Genotype | n | Tyramine (females only) | | | | 4-HPAA (females only) | | | |
| --- | --- | --- | --- | --- | --- | --- | --- | --- | --- | --- |
|  |  |  | **AUC_inf_** (µM*min) | **SD** | **Ae_0-6h_**  (µmol) | **SD** | **AUC_inf_** (mM*min) | **SD** | **Ae_0-6h_** (mmol) | **SD** |
| VNTR | 3/3 | 5 | 23.9 | 3.33 | 5.62 | 1.45 | 9.53 | 1.06 | 2.22 | 0.22 |
|  | 3/4 | 29 | 29.7 | 13.6 | 7.15 | 3.14 | 9.19 | 1.76 | 2.15 | 0.27 |
|  | 4/4 | 20 | 29.1 | 15.7 | 7.32 | 4.49 | 8.80 | 1.55 | 1.96 | 0.66 |
| rs2064070 | T/T | 22 | 31.7 | 16.0 | 7.50 | 4.20 | 8.89 | 1.58 | 2.02 | 0.63 |
|  | TA | 28 | 27.6 | 12.7 | 6.96 | 3.27 | 9.20 | 1.76 | 2.11 | 0.30 |
|  | A/A | 4 | 24.0 | 3.5 | 5.49 | 1.44 | 9.24 | 1.07 | 2.27 | 0.20 |
| rs6323 | T/T | 22 | 31.7 | 16.0 | 7.50 | 4.20 | 8.89 | 1.58 | 2.02 | 0.63 |
|  | GT | 28 | 27.6 | 12.7 | 6.96 | 3.27 | 9.20 | 1.76 | 2.11 | 0.30 |
|  | G/G | 4 | 24.0 | 3.5 | 5.49 | 1.44 | 9.24 | 1.07 | 2.27 | 0.20 |
| rs1137070 | C/C | 22 | 31.7 | 16.0 | 7.50 | 4.20 | 8.89 | 1.58 | 2.02 | 0.63 |
|  | CT | 28 | 27.6 | 12.7 | 6.96 | 3.27 | 9.20 | 1.76 | 2.11 | 0.30 |
|  | T/T | 4 | 24.0 | 3.5 | 5.49 | 1.44 | 9.24 | 1.07 | 2.27 | 0.20 |
| rs909525 | C/C | 20 | 30.1 | 14.8 | 7.39 | 4.40 | 8.84 | 1.56 | 1.98 | 0.65 |
|  | CT | 29 | 29.1 | 14.3 | 7.13 | 3.22 | 9.22 | 1.78 | 2.13 | 0.31 |
|  | T/T | 5 | 23.7 | 3.15 | 5.48 | 1.25 | 9.14 | 0.95 | 2.25 | 0.18 |
| rs2072743 | A/A | 20 | 29.1 | 15.7 | 7.32 | 4.49 | 8.80 | 1.55 | 1.96 | 0.66 |
|  | GA | 30 | 29.6 | 13.4 | 7.16 | 3.09 | 9.23 | 1.75 | 2.14 | 0.27 |
|  | G/G | 4 | 23.3 | 3.5 | 5.15 | 1.16 | 9.28 | 1.04 | 2.29 | 0.18 |
| rs1800464 | A/A | 51 | 28.4 | 13.2 | 7.05 | 3.67 | 9.07 | 1.64 | 2.07 | 0.47 |
|  | CA | 3 | 38.8 | 23.8 | 7.51 | 1.80 | 9.13 | 1.73 | 2.35 | 0.19 |
|  | C/C | 0 |  |  |  |  |  |  |  |  |
| rs1799835 | A/A | 54 | 29.0 | 13.8 | 7.07 | 3.58 | 9.08 | 1.63 | 2.09 | 0.46 |
|  | AC | 0 |  |  |  |  |  |  |  |  |
|  | C/C | 0 |  |  |  |  |  |  |  |  |
| CA-repeat | 17/18 | 14 | 32.2 | 16.4 | 7.73 | 4.89 | 8.88 | 1.63 | 1.88 | 0.76 |
|  | 17/19 | 1 | 11.4 |  | 4.93 |  | 6.50 |  | 2.20 |  |
|  | 18/18 | 8 | 26.9 | 11.4 | 7.08 | 2.29 | 8.74 | 1.79 | 2.16 | 0.24 |
|  | 18/19 | 7 | 30.7 | 18.7 | 7.19 | 3.33 | 9.12 | 1.41 | 2.22 | 0.33 |
|  | 18/21 | 1 | 25.2 |  | 6.80 |  | 8.57 |  | 2.11 |  |
|  | 18/22 | 17 | 29.5 | 13.3 | 6.92 | 3.80 | 9.76 | 1.64 | 2.12 | 0.30 |
|  | 19/20 | 1 | 24.8 |  | 8.94 |  | 7.62 |  | 2.02 |  |
|  | 19/22 | 2 | 24.5 | 3.4 | 6.53 | 1.55 | 7.57 | 1.63 | 2.00 | 0.25 |
|  | 22/23 | 1 | 28.3 |  | 5.76 |  | 9.16 |  | 2.54 |  |
|  | 22/24 | 2 | 21.4 | 1.9 | 4.71 | 1.78 | 9.62 | 1.64 | 2.22 | 0.13 |
| Total | Females only | 54 | 29.0 | 13.8 | 7.07 | 3.58 | 9.08 | 1.63 | 2.09 | 0.46 |

| Active alleles | CYP2D6 genotype | n | Tyramine | | | | 4-HPAA | | | |
| --- | --- | --- | --- | --- | --- | --- | --- | --- | --- | --- |
|  |  |  | **AUC_inf_** (µM*min) | **SD** | **Ae_0-6h_**  (µmol) | **SD** | **AUC_inf_** (mM*min) | **SD** | **Ae_0-6h_** (mmol) | **SD** |
| 0.0 | *4/*4 | 5 | 32.3 | 10.5 | 8.80 | 3.75 | 8.73 | 2.05 | 2.12 | 0.27 |
| 0.0 | *6/*6 | 1 | 70.0 |  | 11.1 |  | 8.24 |  | 1.81 |  |
| 0.5 | *4/*9 | 2 | 33.1 | 24.2 | 7.27 | 3.68 | 9.80 | 0.42 | 2.13 | 0.05 |
| 0.5 | *4/*41 | 1 | 28.9 |  | 4.50 |  | 7.62 |  | 2.07 |  |
| 0.5 | *5/*9 | 1 | 22.1 |  | 5.43 |  | 8.72 |  | 2.18 |  |
| 0.5 | *6/*9 | 1 | 22.7 |  | 3.45 |  | 10.8 |  | 2.13 |  |
| 0.5 | *6/*10 | 1 | 28.7 |  | 6.68 |  | 9.15 |  | 2.12 |  |
| 0.5 | *6/*41 | 1 | 40.8 |  | 11.4 |  | 9.86 |  | 2.23 |  |
| 1.0 | *1/*3 | 1 | 38.3 |  | 7.66 |  | 8.22 |  | 2.24 |  |
| 1.0 | *1/*4 | 9 | 29.7 | 17.9 | 7.63 | 3.24 | 8.24 | 1.84 | 2.18 | 0.19 |
| 1.0 | *1/*6 | 2 | 42.3 | 8.1 | 12.2 | 2.4 | 8.05 | 0.79 | 2.17 | 0.07 |
| 1.0 | *2/*4 | 2 | 13.8 | 8.5 | 4.11 | 2.63 | 8.87 | 0.87 | 2.09 | 0.20 |
| 1.0 | *2/*4/*MxN | 1 | 65.4 |  | 15.0 |  | 5.87 |  | 1.86 |  |
| 1.0 | *4/*35 | 5 | 37.0 | 19.4 | 9.34 | 4.51 | 10.3 | 2.2 | 2.11 | 0.24 |
| 1.0 | *4/*35/*MxN | 1 | 24.8 |  | 4.73 |  | 11.6 |  | 2.52 |  |
| 1.5 | *1/*9 | 3 | 34.4 | 20.9 | 9.67 | 7.62 | 7.84 | 1.49 | 2.03 | 0.30 |
| 1.5 | *1/*10 | 2 | 19.5 | 4.4 | 5.58 | 2.61 | 7.19 | 0.10 | 2.05 | 0.29 |
| 1.5 | *1/*41 | 6 | 28.8 | 12.0 | 9.25 | 3.29 | 8.46 | 1.45 | 2.12 | 0.16 |
| 1.5 | *2/*9 | 1 | 47.6 |  | 12.0 |  | 9.14 |  | 2.14 |  |
| 1.5 | *2/*10 | 1 | 23.8 |  | 6.09 |  | 10.1 |  | 1.69 |  |
| 1.5 | *2/*41 | 4 | 31.5 | 14.0 | 11.9 | 5.8 | 10.0 | 3.0 | 2.67 | 0.52 |
| 1.5 | *10/*35 | 1 | 28.1 |  | 9.96 |  | 8.57 |  | 1.86 |  |
| 1.5 | *35/*41 | 2 | 23.7 | 17.9 | 8.36 | 4.22 | 7.59 | 0.53 | 2.26 | 0.06 |
| 2.0 | *1/*1 | 9 | 25.8 | 13.5 | 5.91 | 2.25 | 8.87 | 1.01 | 2.14 | 0.18 |
| 2.0 | *1/*2 | 7 | 30.8 | 14.7 | 7.35 | 3.83 | 9.34 | 2.35 | 2.21 | 0.55 |
| 2.0 | *1/*5 | 4 | 33.4 | 12.9 | 7.18 | 3.29 | 7.87 | 1.67 | 1.81 | 0.41 |
| 2.0 | *2/*2 | 3 | 15.5 | 12.0 | 4.13 | 4.03 | 8.20 | 0.52 | 1.22 | 1.13 |
| 2.0 | *2/*35 | 4 | 34.7 | 20.1 | 10.5 | 6.6 | 7.69 | 1.61 | 2.31 | 0.24 |
| 2.0 | *35/*35 | 2 | 31.1 | 2.8 | 7.68 | 0.09 | 8.58 | 1.71 | 2.10 | 0.03 |
| 2.5 | *2/*9/*MxN | 1 | 32.0 |  | 6.61 |  | 7.44 |  | 2.04 |  |
| 3.0 | *1/*MxN | 2 | 29.9 | 4.3 | 6.23 | 1.99 | 7.55 | 1.60 | 1.77 | 0.08 |
| 3.0 | *1/*2/*MxN | 1 | 25.2 |  | 6.80 |  | 8.57 |  | 2.11 |  |
| 3.0 | *1/*35/*MxN | 1 | 48.8 |  | 17.6 |  | 9.49 |  | 2.38 |  |
| Total |  | 88 | 30.9 | 14.7 | 8.08 | 4.02 | 8.68 | 1.68 | 2.11 | 0.38 |

| Active alleles | OCT1 gentype | n | Tyramine | | | | 4-HPAA | | | |
| --- | --- | --- | --- | --- | --- | --- | --- | --- | --- | --- |
|  |  |  | **AUC_inf_** (µM*min) | **SD** | **Ae_0-6h_**  (µmol) | **SD** | **AUC_inf_** (mM*min) | **SD** | **Ae_0-6h_** (mmol) | **SD** |
| 0 | H2/H2 | 4 | 22.0 | 6.6 | 6.0 | 1.1 | 8.3 | 1.3 | 2.0 | 0.2 |
| 0 | H2/H3 | 4 | 36.0 | 12.3 | 9.3 | 1.5 | 9.7 | 2.0 | 2.2 | 0.2 |
| 0 | H3/H3 | 2 | 27.5 | 7.7 | 5.1 | 0.4 | 8.7 | 0.0 | 1.9 | 0.3 |
| 0 | H3/H4 | 1 | 37.2 |  | 9.2 |  | 6.2 |  | 1.9 |  |
| 0 | H3/H5 | 1 | 22.6 |  | 7.4 |  | 7.1 |  | 2.3 |  |
| 1 | H1/H2 | 17 | 29.0 | 12.0 | 7.7 | 4.4 | 9.0 | 2.3 | 2.2 | 0.2 |
| 1 | H1/H3 | 10 | 28.6 | 18.4 | 8.5 | 4.6 | 8.6 | 2.2 | 2.2 | 0.5 |
| 1 | H1/H4 | 2 | 28.8 | 8.5 | 7.5 | 4.2 | 8.7 | 0.8 | 2.1 | 0.0 |
| 1 | H1/H5 | 3 | 27.0 | 20.2 | 7.6 | 6.6 | 9.3 | 1.8 | 2.1 | 0.0 |
| 2 | H1/H1 | 44 | 33.0 | 16.0 | 8.4 | 4.1 | 8.6 | 1.4 | 2.1 | 0.5 |
| Total |  | 88 | 30.9 | 14.7 | 8.1 | 4.0 | 8.7 | 1.7 | 2.1 | 0.4 |
